# Editorial Note: Bradykinin B_2_ Receptors of Dendritic Cells, Acting as Sensors of Kinins Proteolytically Released by Trypanosoma cruzi, Are Critical for the Development of Protective Type-1 Responses

**DOI:** 10.1371/journal.ppat.1012070

**Published:** 2024-03-11

**Authors:** The PLOS Pathogens Editors

Concerns have been raised about two FACS panels in Figure 5 of this article [1]. Specifically, the top right and bottom right Heart 28 d pi. B_2_R^-/-^ panels (CD44/IFN-γ) appear highly similar although they are stated to represent two independent replicates.

In response, the authors stated that the Figure 5 legend was inaccurate but the results are correctly reported. As is explained further below, the authors stated that the panels in question represent different technical replicates from two tubes originating from the same biological sample. They commented, “*As flow cytometry is a single-cell analytic tool*, *FACS plots generated from two independent replicates from the same biological sample should yield very similar plots*, *but not necessarily identical*.”

Two members of the Editorial Board reviewed the concerns and authors’ responses, and advised that the degree of similarity between the two panels exceeds what would be expected for different replicates, even if they originated from the same biological sample.

The authors stated that the raw data files used to generate Figure 5 were lost, and so this issue cannot be resolved.

The *PLOS Pathogens* Editors issue this Editorial Note to inform readers of this issue. While we remain concerned about the similarities between the two plots, we also concluded that the article’s overall results and conclusions are not significantly affected since the FACs profiles in question were intended to show two examples of the same experimental result.

In discussing this issue, the authors provided the following methodological clarifications about the experiment reported in Figure 5:

Heart-derived leucocytes from both mice strains were split into two separate tubes, one incubated with the mAb CD4-FITC and the other incubated with mAb CD8-FITC; both tubes were, in parallel, stained with the same antibodies reactive to CD44 (conjugated to the APC fluorochrome) and to IFN-γ (conjugated to the PE fluorochrome). Congruent to data published by independent groups [2], heart tissues of T. cruzi-infected B_2_R^+/+^ mice were infiltrated by CD4^+^ and CD8^+^ T lymphocytes. In contrast, reduced frequencies of CD4^+^ and CD8^+^ T cells were found in infected heart tissues from B_2_RKO mice. Focusing on IFN-γ production, heart tissues from T. cruzi-infected B_2_R^+/+^ mice were confirmed to display CD4^+^ and CD8^+^ T lymphocytes producing IFN-γ. By adding the aforementioned APC anti-CD44 and PE-anti-IFN- γ to separate tubes, frequencies of CD44^+^CD4^+^ T cells and CD44^+^CD8^+^ T cells were then measured in heart tissues of T. cruzi-infected WT mice. Since intracardiac CD4^+^ or CD8^+^ T lymphocytes were hardly detectable in infected B_2_R^-/-^ mice, there was no justification to analyze both CD44 and IFN- γ expression by these classical effector lymphocyte subsets. To exclude the possibility that infected heart tissues from B_2_R^-/-^ mice could have been infiltrated by alternative CD44 leukocyte populations (such as B lymphocytes and T cells) producing IFN- γ, samples from both tubes (originally stained with CD4FITC or CD8FITC) were next treated with APC-anti-CD44 and PE anti-IFN- γ. As shown at the right side of Figure 5, these two plots, gated based on the lymphocyte morphology (FSC x SSC), were nearly identical: hearts from B_2_R-deficient mice lacked IFN- γ -expressing CD44, irrespective of the tube origin (whether treated with FITC-CD4 or FITC-CD8).

In addition, there is an error in the bar chart shown at the bottom of Figure 5: the x-axis label should be % of IFN-g^+^ T cells and not % CD44^+^ IFN-g^+^ T cells.
